# Optimising Cognitive Enhancement: Systematic Assessment of the Effects of tDCS Duration in Older Adults

**DOI:** 10.3390/brainsci10050304

**Published:** 2020-05-16

**Authors:** Claire J. Hanley, Sophie L. Alderman, Elinor Clemence

**Affiliations:** Department of Psychology, Swansea University, Singleton Park, Swansea SA2 8PP, UK; sophiealderman24@gmail.com (S.L.A.); elinorclemence@gmail.com (E.C.)

**Keywords:** transcranial direct current stimulation, non-invasive brain stimulation, stimulation duration, aging, neural plasticity, attentional control

## Abstract

Transcranial direct current stimulation (tDCS) has been shown to support cognition and brain function in older adults. However, there is an absence of research specifically designed to determine optimal stimulation protocols, and much of what is known about subtle distinctions in tDCS parameters is based on young adult data. As the first systematic exploration targeting older adults, this study aimed to provide insight into the effects of variations in stimulation duration. Anodal stimulation of 10 and 20 min, as well as a sham-control variant, was administered to dorsolateral prefrontal cortex. Stimulation effects were assessed in relation to a novel attentional control task. Ten minutes of anodal stimulation significantly improved task-switching speed from baseline, contrary to the sham-control and 20 min variants. The findings represent a crucial step forwards for methods development, and the refinement of stimulation to enhance executive function in the ageing population.

## 1. Introduction

Age-related neurochemical, structural, and functional brain changes are most pronounced in prefrontal regions and produce deficits in response inhibition [1,2], which drastically impact daily living, limiting personal safety, independence, and quality of life [3,4,5]. Such concerns represent a prominent societal challenge as life expectancy increases [6,7]. As pharmacological interventions have been largely ineffective [8,9,10,11], it is imperative that innovative strategies are developed to reduce the incidence of cognitive deficits. 

In recent years, transcranial direct current stimulation (tDCS) has gained interest as a non-invasive and cost-effective method of enhancing cognition, due to its observed neuromodulatory effects on plasticity [12,13,14], particularly deficient neurotransmission [15], which is reported to underlie the presence of cognitive decline on neuropsychological tests [16]. Consequently, the existing evidence signals that the use of tDCS would be highly advantageous in minimising executive deficits. The vast majority of studies have focused on aspects of memory, where some success in enhancing the efficiency of working memory has been described in cognitively healthy older adults [17,18,19]. However, the comparatively limited literature on attentional control means mixed results are even more difficult to interpret [20,21,22]. This discrepancy may be accounted for by subtle variations in stimulation protocols, such that systematic evaluation of individual parameters is necessary to determine optimal results. 

Studies in young adults have highlighted the non-linearity of variations in key stimulation parameters, such as current intensity [23,24,25]. It is not known whether the older population also demonstrate this pattern of results; however, the incidence of age-related brain atrophy likely necessitates the use of distinct protocols, compared to those that are effective in young adults [26,27,28]. Little is known about differences in duration, a crucial variable in relation to the induction of neuroplastic effects [29,30]. A computational modelling study [31] noted reductions in the peak electric fields generated in older adult participants, with the authors suggesting that longer durations of stimulation (than those typically used in conjunction with young adults) may prevent this. Therefore, stimulation of 20 min in length may be ideal where modulations of neuroplasticity are delayed due to diminished integrity of existing mechanisms [32] but, to date, this has not been formally tested.

The aim of this current study was to provide vital insight into the effects of duration for the purpose of further developing the use of tDCS, and refining stimulation protocols, specifically designed for older adults. This was achieved by assessing participants’ task-switching speed, following anodal stimulation of 10 and 20 min, alongside that obtained during a sham-control condition. In line with the consensus in the literature, it was anticipated that task-switching speed would be enhanced after receiving active tDCS for the longer duration. 

## 2. Materials and Methods

### 2.1. Subjects

In total, 40 participants, aged 60–75 years (67.05 ± 5.21, 20 females) were recruited to take part in the study. Prior to recruitment, all participants were asked to complete a screening form. Those with safety screening contraindications were excluded from the study. Contraindications included history of neurological (e.g., seizures, stroke) and/or psychiatric conditions (e.g., anxiety, depression), head trauma, concussion, and surgical implants (e.g., neurostimulator, pacemaker, cochlear implant). Individuals who had been prescribed medication designed to directly influence cortical excitation/inhibition (e.g., gabapentin for nerve pain), which may interfere with the emergence of tDCS effects, were also excluded [33]. All participants had corrected-to-normal vision, and scored in the normal range on the Montreal Cognitive Assessment (MoCA) [34] (27.80 ± 1.18). Participants gave written informed consent prior to taking part in the study. Procedures were carried out with the approval of the local ethics committee (Department of Psychology, Swansea University).

### 2.2. Task-Switching Paradigm

**Figure 1:**
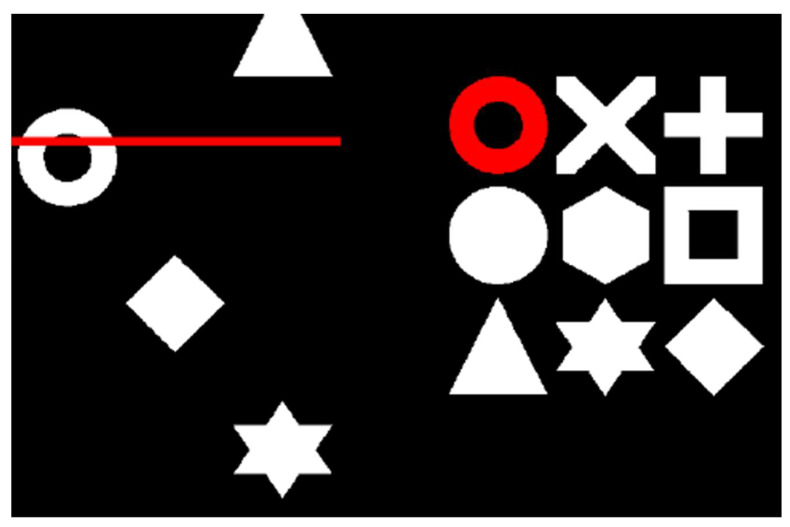
The Swansea Test of Attentional Control (STAC) task. A target is identified within the 3 × 3 matrix of symbols (right). When a matching symbol appears amongst the three columns of the search array that scroll up the screen (left), participants press the spacebar as the symbol crosses behind the red line (as depicted in Hanley and Tales, 2019, [22]).

The task used was identical to that outlined in Hanley and Tales (2019) [22]. The Swansea Test of Attentional Control (STAC) is a complex task-switching paradigm, comprising selective attention, task monitoring, and response inhibition components (Figure 1). Use of a flexible algorithm designed to track performance (Parameter Estimation by Sequential Testing (PEST) [35]) calibrates speed on the basis of prior responses. PEST facilitates completion of the task within the bounds of an individual’s capabilities and ensures that participants are able to respond successfully while not compromising on task difficulty, thereby, making the STAC ideal for use with older adult participants.

Participants were required to remain vigilant throughout the task in order to update the search criteria. The target changed every 12 s, resulting in approximately 25 targets per experimental run of 300 s. Speed (measured in symbols per minute per column; abbreviated to ‘spm’) was adjusted to maintain accuracy around a 75% correct criterion, using the PEST algorithm. Task speed began at 41 spm and increased or decreased in line with accuracy, such that task difficulty corresponded with performance. The participants’ threshold is the speed at which the task is performed at the end of the test (referred to as final speed), whereby higher values represent superior performance.

### 2.3. Transcranial Direct Current Stimulation

With the exception of duration, which was varied in the present study, parameters were identical to those outlined here [22]. Anodal stimulation of 10 and 20 min (1.5 mA), as well as a sham-control variant (10 min), was administered via 25 cm^2^ electrodes positioned in a bihemispheric montage designed to target dorsolateral prefrontal cortex (dlPFC; F3/F4). In line with the available literature [26,27,28], the electrode size was smaller and stimulation intensity was greater than that typically used in conjunction with younger adults, in order to increase the focality of the current and compensate for increases in cerebrospinal fluid (CSF) observed in the ageing brain.

### 2.4. Experimental Procedure

Each participant received the three variants of stimulation (Sham, Active10, Active20) in a counterbalanced order, determined by a random sequence generator, with 7 days between subsequent sessions. Prior to acquiring the baseline data, participants executed the task for approximately 5 target changes to gain experience with the paradigm. Baseline data was acquired prior to stimulation (at the onset of their first session), which was compared to post-stimulation performance measures. Stimulation was administered while participants watched a nature documentary. After stimulation, they were asked to complete an adverse effects questionnaire (AEQ) to determine the presence and severity of stimulation side-effects.

### 2.5. Data Analysis

Data from all 40 participants was entered into statistical analysis using SPSS for Windows software (version 22; IBM, New York). Repeated-measures ANOVAs were used to assess differences relating to the AEQ data across sessions. To identify distinctions in task performance, a one-way, repeated-measures ANOVA was conducted on the STAC final speed data from each acquisition (Baseline, Sham, Active10, Active20). An alpha level of 0.05 was used to determine significance. Bonferroni corrected, post-hoc tests were conducted to investigate the main effect (significant differences from baseline in each of the three experimental conditions, with an adjusted alpha of 0.017). 

## 3. Results

### 3.1. Adverse Effects Questionnaire

Participants reported mild–moderate side effects of stimulation. These reports were consistent across each of the three sessions (producing non-significant differences in tingling, burning, and concentration; *p* > 0.05).

### 3.2. Task-Switching Speed

**Figure 2:**
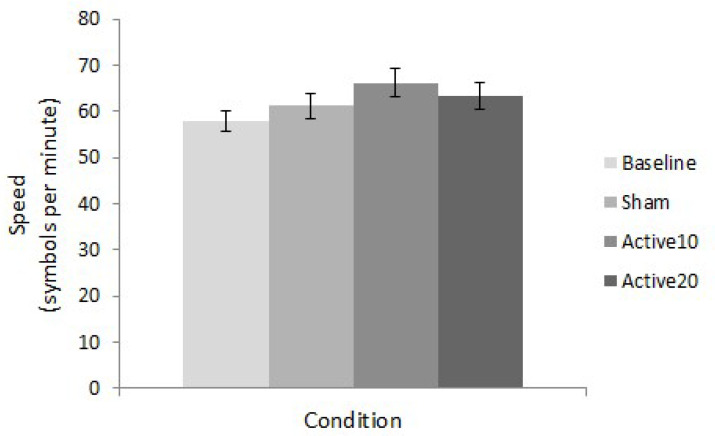
STAC final speed. Mean speed values for all conditions (baseline, sham, Active10, Active20) illustrate superior task performance following 10 min of anodal tDCS. Error bars represent ±1 standard error.

A repeated measures ANOVA revealed a significant difference in task performance across conditions (F(3,117) = 3.016, *p* = 0.033, ηp^2^ = 0.072). Post-hoc t-tests established that this difference was driven by superior task speed in the Active10 compared to baseline condition (t(39) = −4.227, *p* < 0.001) (Figure 2). This result corresponded to a moderate effect size of 0.494 (Cohen’s *d*; see [36]). Comparisons between baseline and sham (t(39) = −1.059, *p* = 0.296) and baseline and Active20 (t(39) = −1.865, *p* = 0.070) conditions were statistically non-significant.

## 4. Discussion

The aim of the present study was to investigate the influence of variations in tDCS parameters as applied to older adults, specifically, by focusing on stimulation duration. When compared to the baseline condition, task-switching speed was significantly enhanced following 10 min of active stimulation; a result which assists in strengthening the limited evidence base in favour of using tDCS to enhance attentional control [21,22]. Neurochemical and/or functional imaging measures would be required to confirm the neurobiological underpinnings of the effect; however, in line with the dominant explanation for tDCS-induced enhancements, it is speculated that performance was facilitated by improved prefrontal network connectivity via the modification of NMDA/GABA receptor response, essential for promoting synaptic plasticity [29,30,37]. Where previous research has failed to establish desirable modulations of attentional control in older adults [20], this may be due to a lack of consideration of such neurobiological mechanisms. Accordingly, the aforementioned study by Boggio et al. directly replicated a tDCS protocol designed for young adults with an older adult sample. While the authors state that this decision stemmed from the aim of comparing performance, it nonetheless highlights a lack of appropriate study design where the populations in question inevitably differ in relation to key neural characteristics. In contrast, in the present study, the selected parameters enhance the biological plausibility of the rationale [38], by reflecting knowledge of age-related brain changes in the context of stimulation [26,27,28].

At the onset of the study, it was predicted that active stimulation, of 20 min in length, would be required to enhance task-switching ability. Conversely, 10 min of anodal tDCS significantly improved STAC final speed, thus challenging the suggestion that longer durations of stimulation are necessary to improve cognition in older adults [27,31,39,40,41]. To date, the limited available literature demonstrates the emergence of cognitive enhancement following 15+ min of active tDCS [13,42,43]. Such stimulation durations are said to compensate for excess CSF, characteristic of the ageing brain, which has been reported to reduce the focality of the current [26,28]. This includes our previous work that adopted a 20 min stimulation protocol, in which improved task-switching speed was established after three subsequent sessions [22]. Therefore, the observation of a single session improvement is equally intriguing given findings of delayed neuroplastic effects in older adults [32], which we had presumed would largely prevent this population from demonstrating an acute response to stimulation. 

Intra-individual variability and non-linear responses to stimulation may account for the emergence of a significant effect at 10 min [44,45]. Accordingly, subtle changes in protocols can have marked effects on the resulting outcomes, hence the need for systematic evaluation of parameters. This implies that individuals have an optimal threshold, attributed to homeostatic constraints on neurobiological circuits to prevent over-excitation of calcium channels and NMDA receptors [46,47]. This effect is readily observed where stimulation is delivered at various intensities [48,49]. These studies demonstrate reliability between subsequent repeats of the same protocol yet assert that higher current strengths are not always necessary to produce modulations of excitability. Similarly, stimulation that is insufficient to fulfil an individual’s optimal threshold may propagate deficient calcium transmission. For example, while increased intracellular calcium is integral for LTP, exceeding optimal levels will activate potassium channels and induce hyperpolarisation, forcing the cell population into a state of LTD or the so called ‘no man’s land’ [50]. This is likely to result in the abolishment of expected neuromodulatory effects [46,51]. Furthermore, in the context of cathodal stimulation, typical inhibitory effects have been shown to be reversed, generating excitation at heightened intensities due to excessive stimulation and habituation of potassium channel response [23]. Cathodal stimulation is likely to diminish performance in older adults in contexts where the anodal polarity has been shown to be successful [52], and performance enhancement was a key objective of the present research. However, it would be interesting to determine whether an equivalent pattern of performance could be produced following inhibitory stimulation, which could potentially aid our interpretation of results.

Given the similarity in methodology, it is not likely that the task or elements of the stimulation protocol (beyond duration) contributed to the distinction between our studies, with regard to the generation of a significant effect following a single session of tDCS. Instead, subtle differences in the samples may account for the disparity in findings. Older adults are a particularly heterogeneous group and individual differences in tDCS response, like those found in conjunction with other non-invasive stimulation methods, are projected to account for approximately 40–50% of variance in outcomes [53,54]. Factors such as genetic variance (e.g., in relation to the regulation of plasticity; Brain-Derived Neurotrophic Factor (BDNF)) may be particularly relevant in the context of older adults, as those who are Val66Met carriers have been established to achieve maximal benefits following longer stimulation durations (20 compared to 10 min) [55]. The Val66Met polymorphism has been linked to a reduction in glutamatergic transmission [56,57], such that longer stimulation durations are required to induce neuroplastic effects. Therefore, variations in the capacity of an individual to modulate plasticity may have profound effects on stimulation outcomes. 

Plasticity is known to decline with age [58] and, for this reason, it is likely desirable to keep age ranges fairly narrow when conducting tDCS research with older adults. This may be an additional reason why some stimulation studies fail to establish beneficial effects in the ageing population (for example [20], in which participants ranged from 50 to 85 years). Between our studies, there was a slight difference in age range, whereby our previous study recruited individuals aged 54–75, compared to 60–75 years in this instance, but both sets of participants had a similar average age (66.5 and 67 years, respectively). Therefore, age *per se* is unlikely to have been a defining factor. Furthermore, average MoCA scores between cohorts were also similarly high (28.2 and 27.8, respectively), signalling that variation in general cognitive function was also an unlikely cause. It is important to note, however, that MoCA score is not directly related to the incidence of frontal atrophy as may be expected [16], suggesting that identical test scores do not equate to similar patterns of atrophy. Distinctions in brain anatomy are likely independent of neuropsychological test outcome; such that where samples perform equally well on a standard cognitive measure, this does not mean they are identical from the perspective of neural change. Consequently, variation in results may be attributed to individual differences in brain structure and function that are commonly associated with older adults [59,60,61].

Anecdotally, many of the participants in the present study were still in employment and reported engaging in regular physical activity, lifestyle factors that mediate age-related decline in grey matter volume and white matter integrity [62,63]. The fact that repeated stimulation worked in the context of the previous study suggests the incidence of greater neural changes, explaining the need for lengthy stimulation, across multiple sessions, in order to alter plasticity and resulting cognitive performance [32]. Given the presence of a more ‘youth-like’ sample than that previously recruited, the neuroplastic mechanisms we sought to strengthen with tDCS may have still been largely intact in the present group, hence why they benefitted from a single session protocol. Without individual anatomical data, we are unable to confirm these differences in neuroanatomy; however, in young adults, long stimulation durations are not necessary to produce cognitive change [23,46]. This is also likely to be the case in the context of older adults, who recruit typical patterns of brain activity and still display hemispheric specialisation [59,60,61]. We intend to investigate this in the future by profiling participants in relation to several structural and functional neuroimaging metrics, as well as individual differences in lifestyle factors, because the integrity of the brain could be key in establishing the optimal duration of stimulation. 

With the acquisition of neuroimaging data, computational modelling would also be possible, similar to that which has established changes in the effects of stimulation in the context of increased CSF [26,28]. A recent study has produced additional evidence to suggest that patterns of atrophy contribute to the amount of current reaching the cortex [64], which highlights the need for further systematic evaluations of approaches to compensate for such shortcomings (e.g., incrementally increasing the intensity of stimulation). Ultimately, generating a biologically plausible forward model to establish the likely outcome of stimulation, given the neuroanatomical status of an individual, could prove to be an incredibly valuable way of enhancing the validity of subsequent research [65]. Specifically, such a model could assist in the development of stimulation protocols to enhance cognition in older adults by providing crucial insight into optimal intensity and advantageous electrode positioning [66,67,68]. 

Incorporating online stimulation, during the task, may also enhance the effectiveness of tDCS. Meta-analyses highlight the benefits of online protocols in older adults due to age-related deficits in plasticity induction [69,70] (although this may largely apply to the motor domain, as opposed to cognition [41]). Nonetheless, it may be advantageous to isolate potential differences in the state-dependency of effects. While such meta-analyses converge on the consensus that tDCS is able to benefit cognitive performance, there is divergence between subtypes, such that it would be useful for studies to be able to compare across domains. This current study was designed to provide further insight into performance enhancement in the under-represented area of attentional control; however, incorporating a working memory task into the procedure would have allowed for a comparison of the findings to a wider range of past literature. In future, an N-back task [71] could act as a valid control measure, for example, because cognitive load can be modulated to parallel the complexity of the STAC. Such an addition would provide the basis for cross-domain inferences on the potential for global cognitive benefits, which could translate to improved function in aspects of daily life [72].

Lastly, with regard to methodological limitations of the stimulation protocol, it should be noted that the single sham session of 10 min prevented complete blinding. For this reason, the study is regarded as a ‘partial blind’ because, while the 20 min stimulation would have been discernible, the nature of the two 10 min sessions was unknown (to both participants and the researchers), as codes were used to execute stimulation. Although differences in duration are likely more obvious, participants can detect subtleties in current strength (particularly where higher intensities are used [73]), yet researchers commonly use a single sham session in the context of systematic investigations of intensity [19,23,24]. This is most likely due to the already high number of sessions required to conduct systematic evaluation studies (both an inherent strength and weakness of a within-subject experimental design), which focus on the influence of variations in active stimulation. One particular study has used this rationale to omit a sham control condition altogether [44]. While this is likely not advisable, the consensus remains that no specific approach to sham stimulation appears to be any more rigorous than another (including repeated sham conditions) [74]. Evidently, there is still much to be learned about the intricacies of control stimulation, particularly in the context of the older adult population.

In conclusion, advances in our understanding of tDCS effects in the context of older adulthood are very much dependent on methodological development and continued research. These results attest to the safety and tolerability of tDCS in older adults [75] and provide a framework within which to continue testing existing mechanistic assumptions, relating to key parameters, and build momentum in advancing towards flexible and feasible strategies to target age-related changes in cognition. Where this can be achieved, progress towards maintaining executive function in the ageing population is likely to translate to respective benefits in tasks of daily function, an increasingly important consideration as life expectancy continues to rise.
